# Pre-differentiation of human neural stem cells into GABAergic neurons prior to transplant results in greater repopulation of the damaged brain and accelerates functional recovery after transient ischemic stroke

**DOI:** 10.1186/s13287-015-0175-1

**Published:** 2015-09-29

**Authors:** Hima C. S. Abeysinghe, Laita Bokhari, Anita Quigley, Mahesh Choolani, Jerry Chan, Gregory J. Dusting, Jeremy M. Crook, Nao R. Kobayashi, Carli L. Roulston

**Affiliations:** Neurotrauma Research Team, Department of Medicine, University of Melbourne, Level 4, Clinical Sciences Building, 29 Regent Street, Fitzroy, VIC 3065 Australia; Department of Surgery, University of Melbourne, Melbourne, VIC Australia; Department of Biochemistry and Molecular Biology, Bio21 Molecular Science and Biotechnology Institute, University of Melbourne, Melbourne, VIC Australia; ARC Centre of Excellence for Electromaterials Science, Intelligent Polymer Research Institute, AIIM Facility, Innovation Campus, University of Wollongong, Squires Way, Fairy Meadow, NSW 2519 Australia; Department of Obstetrics and Gynecology, National University of Singapore, Singapore, Singapore; Cytoprotection Pharmacology Program, Centre for Eye Research, The Royal Eye and Ear Hospital Melbourne, Melbourne, VIC Australia; Department of Opthamology, Faculty of Medicine, University of Melbourne, Melbourne, VIC Australia; Illawarra Health and Medical Research Institute, University of Wollongong, Wollongong, NSW 2522 Australia

## Abstract

**Introduction:**

Despite attempts to prevent brain injury during the hyperacute phase of stroke, most sufferers end up with significant neuronal loss and functional deficits. The use of cell-based therapies to recover the injured brain offers new hope. In the current study, we employed human neural stem cells (hNSCs) isolated from subventricular zone (SVZ), and directed their differentiation into GABAergic neurons followed by transplantation to ischemic brain.

**Methods:**

Pre-differentiated GABAergic neurons, undifferentiated SVZ-hNSCs or media alone were stereotaxically transplanted into the rat brain (n=7/group) 7 days after endothelin-1 induced stroke. Neurological outcome was assessed by neurological deficit scores and the cylinder test. Transplanted cell survival, cellular phenotype and maturation were assessed using immunohistochemistry and confocal microscopy.

**Results:**

Behavioral assessments revealed accelerated improvements in motor function 7 days post-transplant in rats treated with pre-differentiated GABAergic cells in comparison to media alone and undifferentiated hNSC treated groups. Histopathology 28 days-post transplant indicated that pre-differentiated cells maintained their GABAergic neuronal phenotype, showed evidence of synaptogenesis and up-regulated expression of both GABA and calcium signaling proteins associated with neurotransmission. Rats treated with pre-differentiated cells also showed increased neurogenic activity within the SVZ at 28 days, suggesting an additional trophic role of these GABAergic cells. In contrast, undifferentiated SVZ-hNSCs predominantly differentiated into GFAP-positive astrocytes and appeared to be incorporated into the glial scar.

**Conclusion:**

Our study is the first to show enhanced exogenous repopulation of a neuronal phenotype after stroke using techniques aimed at GABAergic cell induction prior to delivery that resulted in accelerated and improved functional recovery.

## Introduction

Stroke is an acute cerebrovascular disorder that remains a major cause of death and disability in the industrialized world [1]. Aside from thrombolysis, which is limited by a narrow therapeutic window, there is no effective therapeutic treatment proven to promote neurological recovery in the postischemic phase [2].

Regenerative events initiated following brain damage are active for weeks following stroke [3, 4], which potentially provides a second window for treatment. Although there are promising treatment strategies that target brain regeneration, including repetitive training, exercise, and physical therapy [5–7], many stroke survivors are often not able to participate in rehabilitation programs until many weeks after a stroke event and delayed rehabilitation results in worse outcomes [8].

Exogenous cell-based therapies to complement endogenous repair mechanisms are currently being trialed in humans following extensive meta-analysis of over 40 studies reporting significant improvements in function after cell transplantation in ischemic animal models [9–11]. Despite early reports of functional benefits in humans [12, 13], a cellular basis for neurological improvement still remains elusive [14]. Whilst neural cell replacement may be achieved, new research shows that neural stem cells (NSCs) can exert trophic effects through secretion of protein factors which induce change in the host tissue to promote functional improvements [15]. In addition to identifying how these cells work, it is equally important to isolate factors that influence stem cell survival and long-term integration within host tissue.

To date, all preclinical and clinical stem cell transplant studies for the treatment of stroke have been conducted using undifferentiated stem cells [10, 16, 17]. Survival of these cells ranges between 0.5 and 30 % [17–23], and although they are capable of forming neuronal populations after transplant in animal models, cells most predominantly differentiate into astrocytes [16, 17, 20]. Functional recovery in these studies has therefore been suggested to be due to trophic support [19, 24], but this effect has not been well characterized or documented.

Recent reports in other models of neurological disease suggest that predifferentiating stem cells into a neuronal phenotype prior to transplant might be a better approach [25–28]. Indeed, differentiated human neural progenitor cell-derived GABAergic neurons injected into the spinal cord following spinal cord injury result in long-term survival of GABAergic cells that generate glutamic acid decarboxylase, gamma-aminobutyric acid (GABA), and β-III tubulin, resulting in functional improvement [26]. We therefore see value in using a similar approach for promoting recovery after stroke. To this end, we have employed human neural stem cells (hNSCs) isolated from the subventricular zone (SVZ) [29] and directed their differentiation into GABAergic neuronal cells. In the present study we investigated the effect of transplanting undifferentiated SVZ-hNSCs versus predifferentiated SVZ-hNSCs into the rat brain 7 days after stroke. We determined the optimal phenotypic conditions that translate into best outcomes in terms of cell survival, histopathology, and functional recovery.

## Materials and methods

The following experiments were conducted in adherence with current RIGOR guidelines [30, 31] and included randomization of treatments, blinding during assessment, inclusion of appropriate control groups, and full statistical analysis involving power calculations in consultation with the Statistical Consulting Centre, University of Melbourne, Victoria, Australia.

### Ethics statement and animals

All experiments were performed in strict accordance with the guidelines of the National Health & Medical Research Council of Australia Code of Practice for the Care and Use of Animals for Experimental Purposes in Australia. The protocol was approved by the St Vincent’s Hospital animal ethics committee (AEC009/09). Surgeries were performed under general anesthesia and paracetamol (2 mg/kg in drinking water) was provided 24 hours prior to and after surgery to minimize distress. A total of 30 adult male Hooded Wistar rats weighing 300–360 g (Laboratory Animal Services, University of Adelaide, Australia) were used for experiments. Rats were group housed (four rats/cage) prior to stroke, whereupon they were individually housed on a 12-hour light/dark cycle with ad libitum access to food and water.

### Surgical preparation

Rats were anesthetized with a mixture of ketamine/xylazine (75 mg/kg:10 mg/kg respectively intraperitoneally) and maintained throughout surgery by inhalation isoflurane (95 % O_2_ and 5 % isoflurane). A 23-gauge stainless steel guide cannula was stereotaxically implanted into the cortex 2 mm dorsal to the right middle cerebral artery (MCA) (0.2 mm anterior, –5.9 mm lateral, –5.2 mm ventral) as in previous studies [32, 33]. Rats were allowed to recover for 5 days prior to stroke induction.

### Endothelin-1-induced stroke

Focal cerebral ischemia was induced in conscious rats (*n* = 30) by constriction of the right MCA with perivascular administration of endothelin-1 (ET-1, 60 ρmol in 3 μl saline over 10 minutes; American Peptide Company, Inc., CA, USA) [32, 33]. Characteristic behavioral changes indicative of stroke were scored 1–5 for stroke severity according to our previously established protocol, with 5 being the most severe [33]. Rats that did not display behavioral changes were considered not to have suffered a stroke and were excluded from further study. Only rats with an initial stroke severity score of 4, and with clear deficits of ≥3 according to the neurological deficit score at 7 days, were used for stem cell transplants so as to minimize sample variation associated with different stroke outcomes [32, 33].

### In vitro cell preparation

#### hNSC expansion, medium preparation and handling

Experiments were conducted using hNSCs isolated from the SVZ of human fetal brain tissues (14–21 weeks) that were obtained following donor consent under the strict guidelines approved by the Singapore National Healthcare Group Domain Specific Review Board (reference: D/06/154) [29]. The hNSCs were cultured as free-floating neurospheres using protocols described previously.

Prior to transplant, cryopreserved SVZ-hNSCs were thawed, passaged 48 hours later, and expanded as described previously [29]. Briefly, cells were collected and mechanically dissociated by trituration, and approximately 0.7 × 10^6^ cells/well were plated into low-adherence six-well plates (Corning Costar, Sigma-Aldrich, St. Louis, MO, USA). For expansion, cultures were maintained in complete media (Neurocult NS-A hNSC proliferation media and supplement; Stem Cell Technologies Inc., Tullamarine, VIC, Australia), human epidermal growth factor (EGF, 20 ng/ml; Peprotech, Rocky Hill, NJ, USA), human basic fibroblast growth factor (bFGF, 20 ng/ml; Peprotech), heparin (2 μg/ml; Sigma-Aldrich, St. Louis, MO, USA), and 1 % penicillin/streptomycin (Gibco, Thermo Fisher Scientific, Waltham, MA, USA) at 37 °C in a humidified incubator (5 % CO_2_ atmosphere) for a minimum of 14 days to reach appropriate numbers required for differentiation and transplantation. A 50 % media change was performed every second day. Cell density and viability (≥90 %) was determined using the standard 0.1 % trypan blue exclusion test [29].

#### *In vitro* differentiation of hNSCs

For differentiation of SVZ-hNSCs, neurospheres were first dissociated into single-cell suspensions with TryplE, and then cultured in differentiation media consisting of Dulbecco’s modified Eagle’s medium (DMEM)/F12 (Invitrogen, Thermo Fisher Scientific) and neurobasal media (1:1) supplemented with 1 % StemPro neural (Invitrogen), 0.5 % N2 (Gibco), and 50 ng/ml brain-derived neurotrophic factor (BDNF; Peprotech) to induce differentiation as described previously [29]. All cultures were maintained at 37 °C in a humidified culture incubator (5 % CO_2_ atmosphere) for 7 days, with a 50 % differentiation media change every second day.

#### *In vitro* immunocytochemistry

To determine the phenotype of undifferentiated (day 0) cells, SVZ-hNSC neurospheres were seeded onto poly-l-lysine-coated (Sigma-Aldrich, St. Louis, MO, USA) and laminin-coated (Sigma) eight-well chamber slides (Nunc Lab-Tek, Thermo Fisher Scientific) at a density of approximately 200,000 cells/ml. After seeding, for day 0 analysis, neurospheres were allowed to attach for 2 hours at 37 °C under 5 % CO_2_ before removal of media and fixation with 4 % paraformaldehyde (PFA) in 0.1 M phosphate-buffered saline (PBS) for 15 minutes for immunocytochemical analysis. For analysis of differentiated SVZ-hNSCs (day 7), hNSCs were seeded onto poly-l-lysine-coated (Sigma) and laminin-coated (Sigma) eight-well chamber slides as already described. SVZ-hNSCs were placed in differentiation media as described earlier and after 7 days the cells were fixed with 4 % PFA for 10 minutes at room temperature, washed (1 × 5 minutes in 0.1 M PBS), and processed for immunocytochemistry.

Immunofluorescence was performed on in vitro cell culture chamber slides to analyze phenotypic profiles as described previously [26]. Briefly, cells were blocked with 5 % normal goat serum (NGS) (or 1 % bovine serum albumin [BSA; Sigma-Aldrich, St. Louis, MO, USA] for all staining with goat anti-parvalbumin (PV) antibody) in 0.1 % Triton X-100 in 0.1 M PBS for 20 minutes before SVZ-hNSCs were identified using primary antibodies including mouse anti-human specific nuclear antigen (HuNu, 1:1000; Millipore, Billerica, MA, USA), rabbit anti-neuronal class III β-tubulin (Tuj1, 1:1000; Covance Inc., Sydney, NSW, Australia), rabbit anti-GABA (1:1000; Sigma), rabbit anti-Nestin (1:1000; Millipore), rabbit anti-glial fibrillary acidic protein (GFAP, 1:1000; DAKO, Glostrup, Hovedstaden, Denmark), guinea-pig anti-doublecortin (DCX, 1:500; Millipore), rabbit anti-neuronal nuclear antigen (NeuN, 1:1000; Millipore), rabbit anti-Ki67 (1:500; Thermo Fisher Scientific), rabbit anti-SOX2 (1:400; Millipore), rabbit anti-glutamate decarboxylase 65&67 (GAD, 1:1000; Millipore), rabbit anti-synaptophysin (SYN, 1:200; Sigma), chicken Tuj1 (1:200; Millipore), mouse anti-calbindin-D28k (CB, 1:1000; Swant, Marly, Fribourg, Switzerland), goat anti-PV (1:1000; Swant), and rabbit anti-calretinin (CR, 1:1000; Swant). All primary antibodies were diluted in 5 % NGS in 0.1 M PBS and cells were incubated for 2 hours at room temperature followed by washing in 0.1 M PBS.

Secondary fluorophore-conjugated antibodies (1:500 for all) included Alexa-568 goat anti-mouse, Alexa-488 goat anti-mouse, Alexa-568 goat anti-rabbit, Alexa-488 goat anti-rabbit, Alexa-488 goat anti-guinea pig, Alexa-555 goat anti-chicken, Alexa-647 goat anti-chicken, and Alexa-568 donkey anti-goat (Invitrogen). Antibodies were diluted in 5 % NGS in 0.1 M PBS and cells were incubated for 1 hour followed by washing in 0.1 M PBS.

In all experiments, DNA counterstain 4′,6-diamidino-2-phenylindole (DAPI; Molecular Probes, Thermo Fisher Scientific) was applied before coverslipping with ProLong gold anti-fade reagent (Invitrogen). Control experiments included either omission of each primary antibody from the protocol or the inclusion of the appropriate IgG control to verify the specificity of each antibody. Resulting sections were examined with a Nikon confocal laser scanning microscope (Nikon Instruments Inc., Melville, NY, USA).

Stereology for cultured cells was assessed by manually quantifying immunolabeled cells at a magnification of × 60 using the confocal microscope. Cells in two wells were analyzed for each immunocytochemical stain. Cell counts were performed at six randomly selected sites within each culture well. The total number of cells immunoreactive for Tuj1, GABA, Nestin, GFAP, SOX2, and Ki67 was determined and expressed as a percentage of the total number of HuNu-positive cells.

#### Preparation of cells for transplantation

SVZ-hNSCs were prepared for transplantation by treating neurospheres with 0.01 % DNase/DMEM for 20 minutes at 37 °C followed by rinsing in 0.01 % DNase/DMEM and centrifugation at 190 × *g* for 5 minutes, resuspension in 0.01 % DNase/DMEM, and gentle trituration into a single-cell suspension.

SVZ-hNSCs were predifferentiated in preparation for transplantation by culturing neurospheres in the presence of BDNF and neural supplements as described earlier. After 7 days, differentiated SVZ-hNSCs were collected and centrifuged at 190 × *g* for 5 minutes, and a single-cell suspension was made prior to transplantation.

Undifferentiated SVZ-hNSCs or predifferentiated cell suspensions comprised a final cell concentration of 400,000 cells/μl and ≥90 % viability for transplantation.

### Experimental design

#### Surgical transplantation

Seven days after ET-1-induced stroke, rats were re-anesthetized as already described. A total of 800,000 cells in 2 μl were delivered to each graft site via a Hamilton syringe attached to a glass capillary (open diameter 50–70 μm). The glass capillary was left in place for a further 5 minutes after each dose before retrieval. A total of eight graft sites relative to the bregma were chosen across the striatum and cortex as follows: Site 1, 1.4 mm anterior, –4.9 mm lateral, –3 and –5 mm ventral; Site 2, 1.4 mm anterior, –2.8 mm lateral, –3.6 and –5.6 mm ventral; Site 3, 0.2 mm anterior, –3.8 mm lateral, –4.8 and –5.8 mm ventral; and Site 4, –2.16 mm anterior, –6 mm lateral, –3.3 and –4.3 mm ventral. All rats were immunosuppressed by daily intraperitoneal injections of cyclosporine (10 mg/kg intraperitoneally daily; Sandimmune, Novartis Pharmaceuticals, East Hanover, NJ, USA) commencing 2 days prior to cell transplantation.

#### Functional assessments

Neurological assessments were conducted on all rats prior to any surgical procedures (pre surgery), immediately prior to ET-1-induced stroke (0 hours post stroke), 24 and 48 hours post stroke, 7 days after stroke (day 0 post transplant), and again 7 and 28 days post transplant. Forelimb asymmetry was analyzed using a cylinder test and the percentage of forelimb placements made on the wall of the plexiglass cylinder during rearing was determined using the methods of Schallert et al. [34]. Animals were also tested for neurological abnormalities and assigned deficit scores based on abnormal posture and hemiplegia as described previously [32, 33, 35, 36]. All scores from each rat post stroke were compared with prestroke scores, and thus each rat acted as its own control.

### Tissue processing and analysis

Rats were re-anesthetized 28 days post transplant and transcardially perfused with 300 ml ice-cold PBS (0.1 M, pH 7.4) followed by 300 ml cold 4 % PFA (in PBS, 0.1 M, pH 7.4) for 15 minutes. Forebrains were removed and post-fixed overnight in 4 % PFA in 0.1 M PBS at 4 °C followed by cryoprotection overnight in 0.1 M PBS with 30 % sucrose at 4 °C. Brains were then frozen and coronal sections (40 μm) prepared using a cryostat and processed as free-floating tissue sections.

#### In vivo immunohistochemistry

Immunofluorescence staining was performed on tissue sections to analyze and quantify graft survival, maturation, and differentiation as described previously [26]. Briefly, free-floating tissue sections were blocked using standard immunohistochemical techniques with 5 % NGS (or 1 % BSA [Sigma] for all staining with goat anti-PV antibody) in 0.3 % Triton X-100 in 0.1 M PBS before SVZ-hNSCs were identified using primary antibodies including mouse anti-HuNu (1:1000; Millipore), rabbit anti-Tuj1 (1:1000; Covance Inc.), rabbit anti-GABA (1:1000; Sigma), rabbit anti-Nestin (1:1000; Millipore), rabbit anti-GFAP (1:1000; DAKO), rabbit anti-NeuN (1:1000; Millipore), rabbit anti-Ki67 (1:500; Thermo Fisher Scientific), rabbit anti-SOX2 (1:400; Millipore), rabbit anti-GAD (1:1000; Millipore), rabbit anti-SYN (1:200; Sigma), chicken anti-Tuj1 (1:200; Millipore), mouse anti-CB (1:1000; Swant), goat anti-PV (1:1000; Swant), rabbit anti-CR (1:1000; Swant), rabbit anti-cleaved caspase-3 (Casp3, 1:500; Cell Signaling Technology, Boston, MA, USA), and rabbit anti-von Willebrand Factor (vWF, 1:200; Millipore).

Secondary fluorophore-conjugated antibodies (1:500 for all) included Alexa-568 goat anti-mouse, Alexa-488 goat anti-mouse, Alexa-350 goat anti-mouse, Alexa-488 goat anti-rabbit, Alexa-647 goat anti-rabbit, Alexa-555 goat anti-chicken, Alexa-647 goat anti-chicken, and Alexa-568 donkey anti-goat (Invitrogen). Antibodies were diluted in blocking solution.

For all experiments, DNA counterstain DAPI (Molecular Probes, Thermo Fisher Scientific ) was applied before coverslipping with ProLong gold anti-fade reagent (Invitrogen). Control studies included either omission of each primary antibody from the protocol or the inclusion of the appropriate IgG control to verify the specificity of each antibody. Staining for terminal transferase-mediated dUTP nick end-labeling (TUNEL) was performed using a TUNEL staining kit (Promega DeadEnd Colorimetric TUNEL system; Promega, Madison, WI, USA) according to the manufacturer’s instructions. Stained sections were examined with a Nikon confocal laser scanning microscope (Nikon Instruments, Melville, NY, USA). Orthogonal projections were performed using National Institute of Health ImageJ software (NIH).

### Quantification and stereology

#### Infarct assessment

Eight predetermined coronal planes throughout the brain from 3.2 to –6.8 mm relative to the bregma [33] were collected for NeuN staining. Triplicate sections from each level were visualized with an Olympus (Albertslund, Denmark) microscope and stroke-damaged regions were identified as areas with distinct absence of NeuN stain, which was analyzed using ImageJ software (NIH, Bethesda, MD, USA) [37–39]. The infarct volume was determined by integrating the cross-sectional area of damage at each stereotaxic level and the distance between the levels [40], with edema corrected for as described previously [33, 41].

#### Stereological analysis

The optical fractionator stereological method was used to obtain unbiased estimates of the total number of HuNu-positive nuclei within undifferentiated and predifferentiated cell graft sites (*n* = 7/group) using Stereo Investigator software (MBF Bioscience, Williston, VT, USA) [20, 25, 42]. Briefly, every third section was analyzed using a fluorescence microscope (BH-2; Olympus, Tokyo, Japan) equipped with a QIClick scientific camera (QImaging, Surrey, BC, Canada) at a magnification of × 60, where a three-dimensional optical dissector counting probe (*x*, *y*, *z* dimensions of 30 μm × 30 μm × 10 μm respectively) was applied to a systematic random sample of sites within the cell graft [25]. Only HuNu-positive cells with clearly visible fluorescence were quantified.

#### Cell phenotyping

The number of HuNu-positive cells that expressed Tuj1, GABA, Nestin, GFAP, and Ki67 was manually counted for phenotypic analysis of cells in vivo within transplant sites. Cells were considered colocalized if labeling of markers was seen throughout the extent of the HuNu-positive nucleus or if a cytoskeletal/cytoplasmic marker surrounded the HuNu-positive nuclear marker. Quantitative analysis was performed in three tissue sections 280 μm apart per graft site (four graft sites/rat) using *z*-scan confocal microscopy at × 40 magnification for animals that received undifferentiated SVZ-hNSCs or predifferentiated cells (*n* = 7/group) as described previously [43–47]. Briefly, estimates of the total number of cells colocalizing with markers of interest were obtained using the following formula:

$$ E=k{\displaystyle \sum N} $$

where *E* is the estimate of the total number of stained cells in each case, ∑*N* is the sum of *n* values in the three sections analyzed, and *k* indicates that every *k*th section was considered (*k* = 8). *N* was corrected according to Abercrombie’s formula:

$$ N= nt/\left(t+D\right) $$

where *n* is the number of cells counted in each section, *t* is the section thickness, and *D* is the mean diameter of the cells [48]. Colocalized cell counts were expressed as the percentage of the total number of HuNu-positive cells.

Because of the positioning of hNSC grafts, very few sections collected contained the exact same anatomical region within the SVZ for accurate comparisons of host neurogenesis across all rats. For this reason, only a qualitative assessment of the influence of exogenous transplants on host neurogenesis was made.

### Statistical analysis

Data obtained in the cylinder test were analyzed by two-way repeated-measures analysis of variance (ANOVA) followed by the Bonferroni post-hoc test to compare differences between treatment groups over time. Data obtained for infarct area and volume assessments were analyzed by two-way ANOVA followed by the Bonferroni post-hoc test. Neurological deficit scores were analyzed by Kruskal–Wallis nonparametric ANOVA followed by Dunn’s post test. We performed a post-hoc power calculation to arrive at 73 % where *P* <0.01, in which deficits were considered improved by a score of 1 with a standard deviation of 0.87. All cell counts were analyzed by two-way ANOVA followed by the Bonferroni post-hoc test to compare treatment groups and graft sites. Data were analyzed with GraphPad Prism, version 6 (GraphPad Software Inc., San Diego, CA, USA) and presented as mean ± standard error of the mean (SEM). Statistical significance was defined as *P* <0.05.

## Results

### In vitro characterization of cells

**Fig. 1:**
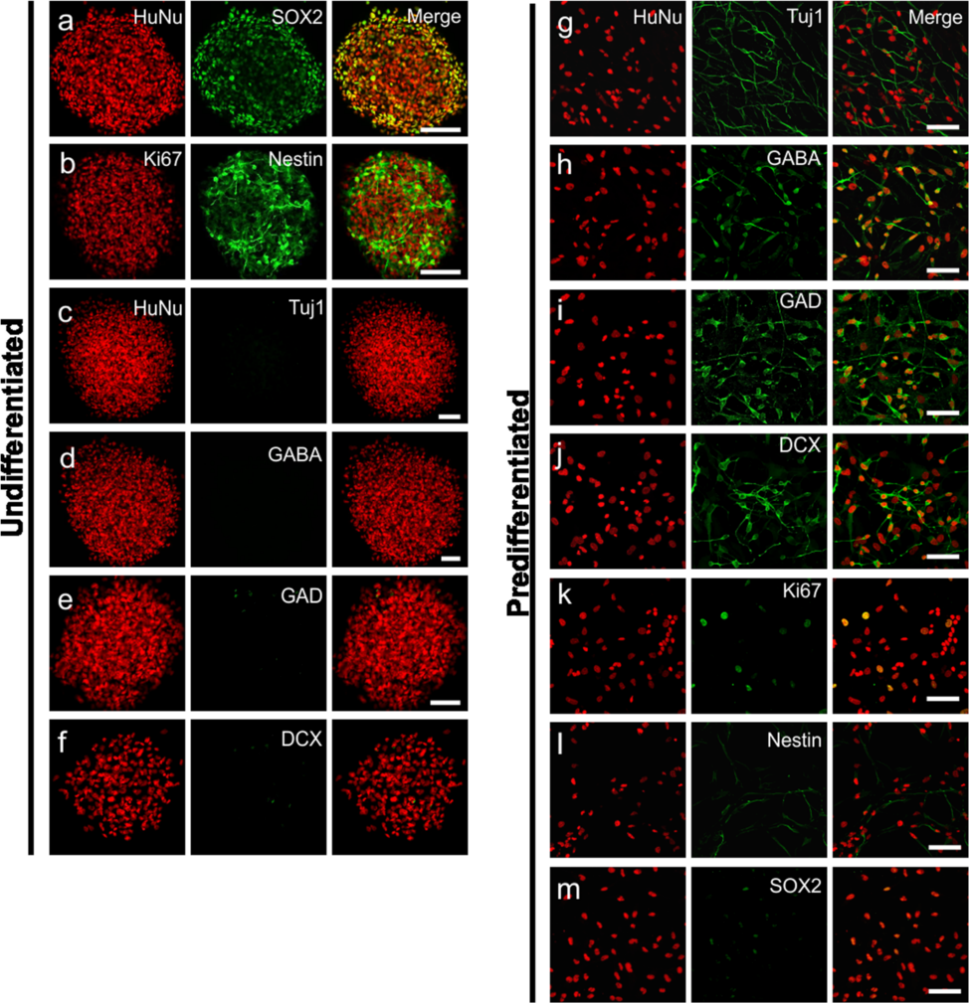
Undifferentiated hNSCs and predifferentiated cell phenotypes in vitro. Immunofluorescent confocal images of undifferentiated SVZ-hNSCs in culture expressed HuNu (*red*) and SOX2 (*green*) (**a**), and Ki67 (*red*) and Nestin (green) (**b**), with colocalization giving a yellow appearance. Undifferentiated SVZ-hNSCs immunopositive for HuNu (*red*) (**c**–**f**) in culture were not observed to express Tuj1 (*green*) (**c**), GABA (*green*) (**d**), GAD (*green*) **e**, or DCX (*green*) **f**. Predifferentiated cells cultured for 7 days demonstrated immunoreactivity for HuNu (*red*) **g**–**m** and colabeled with Tuj1 (*green*) (**g**), GABA (*green*) (**h**), GAD (*green*) (**i**), and DCX (*green*) (**j**). Predifferentiated cells HuNu (*red*) cultured for 7 days demonstrated little immunoreactivity for Ki67 (*green*) (**k**), Nestin (*green*) (**l**), or SOX2 (*green*) (**m**). Scale bar: (**a**–**f**) 100 μm, (**g**–**m**) 50 μm. *DCX* doublecortin, *GABA* gamma-aminobutyric acid, *GAD* glutamate decarboxylase 65&67, *HuNu* human specific nuclear antigen, *Tuj1* β-III tubulin

Confocal analysis of immunolabeled undifferentiated SVZ-hNSCs in vitro revealed formation of neurospheres that were colocalized with HuNu and the undifferentiated cell marker SOX2 (98.9 ± 0.7 %) (Fig. 1a). These cells also colocalized with proliferation marker Ki67 (59.5 ± 3.1 %) and NSC marker Nestin (95.1 ± 0.7 %) (Fig. 1b), but did not express early neuronal marker Tuj1, inhibitory neurotransmitter GABA, GABA-producing enzyme GAD, or immature neuronal marker DCX (Fig. 1c–f). In contrast, predifferentiated SVZ-hNSCs labeled with HuNu were positive for Tuj1 (92.3 ± 1.4 %) (Fig. 1g), GABA (90.1 ± 2.7 %) (Fig. 1h), GAD (Fig. 1i), and DCX (Fig. 1j). Very few predifferentiated cells expressed Ki67 (9.6 ± 2.5 %) (Fig. 1k) or Nestin (28.5 ± 2.6 %, Fig. 1l) and SOX2 (12.1 ± 1.7 %, Fig. 1m). No expression of GFAP, SYN, or the intracellular calcium binding proteins (CBPs) CB, CR, or PV was detected in vitro*.*

### Transplant studies

#### Treatment groups

A total of 21 rats were included in this study. Seven rats were excluded prior to stem cell transplant based on low stroke severity scores and minimal functional deficits detected at 7 days [32, 33]. Only rats with a stroke rating of 4 and a clear functional deficit of ≥3 were randomly assigned to three experimental groups (n = 7/group): Group 1 received media without any cells (vehicle control); Group 2 received undifferentiated SVZ-hNSC transplants; and Group 3 received predifferentiated cells.

#### Functional outcomes

##### Cylinder test

**Fig. 2:**
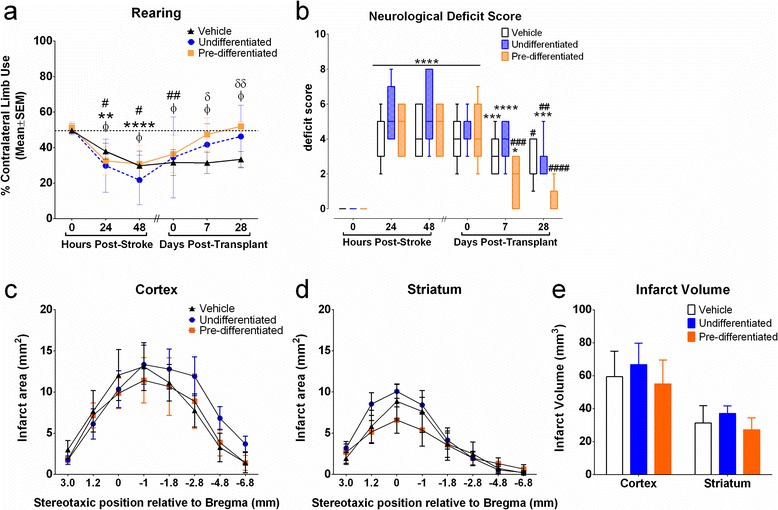
Functional outcome following transplantation. Effects of transplantation on contralateral limb use when rearing (**a**) in the cylinder test after ET-1-induced stroke. Data are mean ± standard error of the mean (SEM) expressed as a percentage of contralateral (impaired) forelimb use. Each rat acted as its own control; results following stroke were compared with 0-hour prestroke scores. ϕ*P* <0.05, ϕϕ*P* <0.01, ϕϕϕϕ*P* <0.0001 relative to 0-hour poststroke baseline scores for vehicle-treated rats (*n* = 7); **P <0.01, ***P <0.001, ****P <0.0001 compared with 0-hour poststroke baseline scores for undifferentiated treated rats (*n* = 7); #*P* <0.05, ##*P* <0.01 relative to 0-hour poststroke baseline scores for predifferentiated treated rats (*n* = 7); δ*P* <0.05, δδ*P* <0.01 vehicle-treated rats compared with predifferentiated treated rats; φφ*P* <0.01 vehicle-treated rats compared with undifferentiated treated rats; †*P* <0.05 predifferentiated treated rats compared with undifferentiated treated rats (two-way ANOVA followed by Bonferroni post test). Combined neurological deficit scores (**b**). Data presented as box plots include hinges extending from the 25th to 75th percentiles, the median line within the box and whiskers extending to the minimum and maximum values of the dataset (*n* = 7/group). *P <0.05, ***P <0.001, ****P <0.0001 relative to 0-hour poststroke baseline scores (*n* = 7/group); #*P* <0.05, ##*P* <0.01, ###*P* <0.001, ####*P* <0.0001 relative to pretransplant scores (Kruskal–Wallis ANOVA followed by Dunn’s test). Effect of transplanting vehicle, undifferentiated hNSCs, or predifferentiated cells on infarct area (**c**), (**d**) and total infarct volume (**e**) within the cortex and striatum. Data presented as mean ± SEM of infarct area measured at eight predetermined coronal planes through the brain (two-way ANOVA followed by Bonferroni post test)

No significant bias in forelimb use upon rearing was detected using the cylinder test prior to stroke induction in any treatment group. After stroke, all rats exhibited asymmetrical limb use indicative of stroke damage with preferential ipsilateral (unimpaired) forelimb use during rearing (Fig. 2a). While transplantation of either undifferentiated SVZ-hNSCs and predifferentiated SVZ-hNSCs appeared to decrease forelimb asymmetry after treatment, this effect was only statistically significant in rats receiving predifferentiated hNSCs at 7 and 28 days post transplant in comparison with vehicle control rats receiving media alone (Fig. 2a) (*P* <0.05, two-way ANOVA).

##### Neurological deficit score

No neurological deficits were observed prior to ET-1-induced stroke. However, significant deficits were observed in all treatment groups between 24 hours and 7 days after stroke (day 0 post transplant) (*P* <0.001, nonparametric ANOVA) (Fig. 2b). By 28 days, rats receiving vehicle control or undifferentiated hNSCs showed significant recovery in their deficits when compared with pretransplant scores (*P* <0.01). Rats that received predifferentiated hNSCs demonstrated earlier recovery with significant improvements observed 7 days post transplant (*P* <0.001) when compared with pretransplant scores, with no significant deficits detected 28 days post transplant when compared with prestroke scores (*P >*0.05).

#### Infarct assessment

Absence of NeuN immunoreactivity revealed stroke-induced damage to the parietal, insular, and frontal cortex, as well as the striatum as reported previously [33]. Both infarct area (Fig. 2c, d) and infarct volume (Fig. 2e) within the cortex and striatum were consistent across treatment groups with no significant differences detected between groups (*P >*0.05, two-way ANOVA).

#### Characterization of cells within grafts

##### Cell counts

**Fig. 3:**
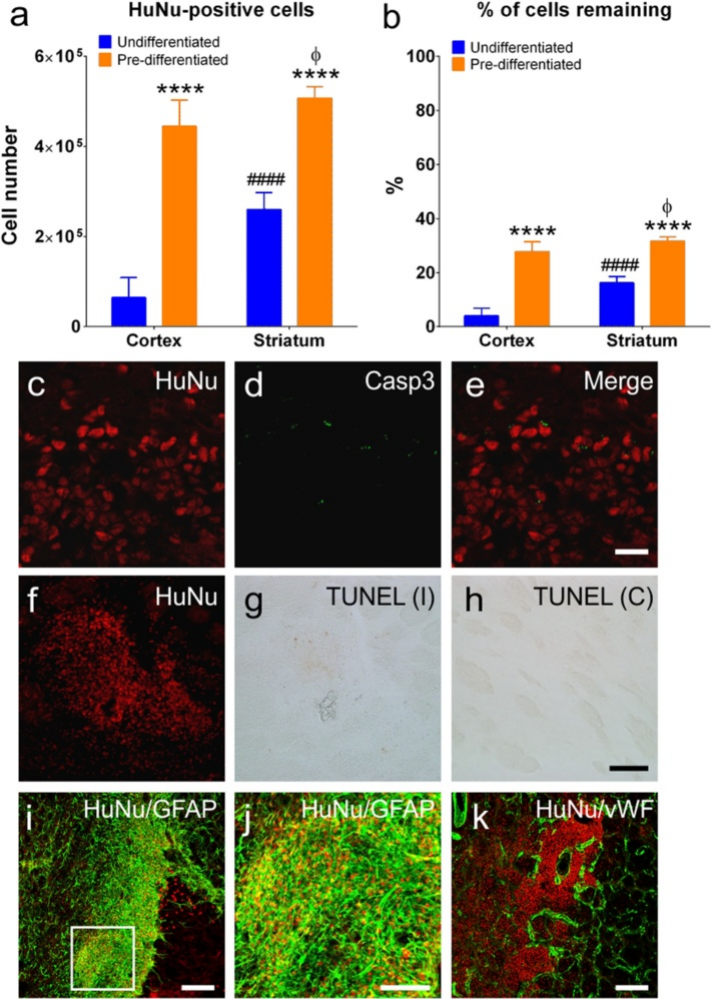
Transplanted cells survive within the stroke affected brain. Total number (**a**) and percentage (**b**) of HuNu-positive cells remaining within cortical and striatal grafts from undifferentiated (*n* = 7) and predifferentiated (*n* = 7) treatment groups. Data presented as mean ± SEM. ****P <0.0001 relative to undifferentiated treated animals in the same region; ####*P* <0.0001 relative to undifferentiated grafts within the cortex; ϕ*P* <0.05 relative to predifferentiated grafts within the striatum (two-way ANOVA followed by Bonferroni post test). Transplanted cells immunopositive for HuNu (*red*) (**c**, **f**) did not express apoptotic markers including Casp3 (*green*) (**d**), merged image (**e**), or TUNEL (**g**), with the level of TUNEL staining similar to the contralateral mirror image (**h**). Transplanted HuNu-positive (*red*) predifferentiated cells were associated with vWF-stained blood vessels (*green*) within infarcted brain regions (**i**). Many HuNu-positive undifferentiated hNSCs (*red*) were found within border regions consisting of GFAP-positive astrocytes (*green*) (**j**). Magnified immunofluorescent image (**k**) corresponds to box highlighted in (**j**) illustrating incorporation of undifferentiated hNSCs into the glial scar bordering the infarct. Scale bar: (**c**–**e**) 20 μm, (**f**–**h**) 100 μm, (**i**, **k**) 200 μm, (**j**) 100 μm. *C* contralateral hemisphere, *Casp3* cleaved caspase-3, *GFAP* glial fibrillary acidic protein, *HuNu* human specific nuclear antigen, *I* ipsilateral hemisphere, *TUNEL* terminal transferase-mediated dUTP nick end-labeling, *vWF* von Willebrand factor

HuNu immunostaining revealed large numbers of SVZ-hNSCs within each graft site situated both within and outside the damaged regions 28 days post transplant. Although rats receiving undifferentiated hNSCs displayed evidence of cells within each transplant site, many HuNu-positive NSCs were also detected beyond the transplant regions and were also visible within the infarct border zone. Rats that received predifferentiated SVZ-hNSCs showed dense clustering of cells within each site. Stereological cell counting revealed significantly fewer undifferentiated SVZ-hNSCs within the cortex and striatum (64,712 ± 16,866 cells and 260,278 ± 14,112 cells respectively) compared with predifferentiated HuNu-positive cells in cortical and striatal graft regions (444,852 ± 22,181 and 508,098 ± 10,031 cells respectively) (*P* <0.0001; Fig. 3a). A greater number of predifferentiated (*P* <0.05) and undifferentiated (*P* <0.0001) SVZ-hNSCs remained within striatal grafts compared with cortical graft sites (Fig. 3a). However the overall percentage of transplanted predifferentiated cells remaining within cortical and striatal graft sites (27.8 ± 1.3 % and 31.8 ± 0.5 % respectively) was significantly higher than that of undifferentiated SVZ-hNSCs within the cortex and striatum (4.0 ± 1.0 % and 16.3 ± 0.9 % respectively) (*P* <0.0001; Fig. 3b).

Survival of transplanted cells within graft sites was confirmed by immunohistochemical analysis for apoptotic marker Casp3 and TUNEL staining. Negligible Casp3 immunostaining (Fig. 3c–e) and TUNEL (Fig. 3f, g) staining was observed within cells 28 days post transplant. The low level of TUNEL staining within the graft was similar to that observed within the contralateral hemisphere (Fig. 3h). Many transplanted undifferentiated SVZ-hNSCs were associated within the glial scar and were GFAP-positive (Fig. 3i, j), whilst transplanted predifferentiated cells were clearly associated with blood vessels within the infarcted regions (Fig. 3k).

##### Cell phenotypes

**Fig. 4:**
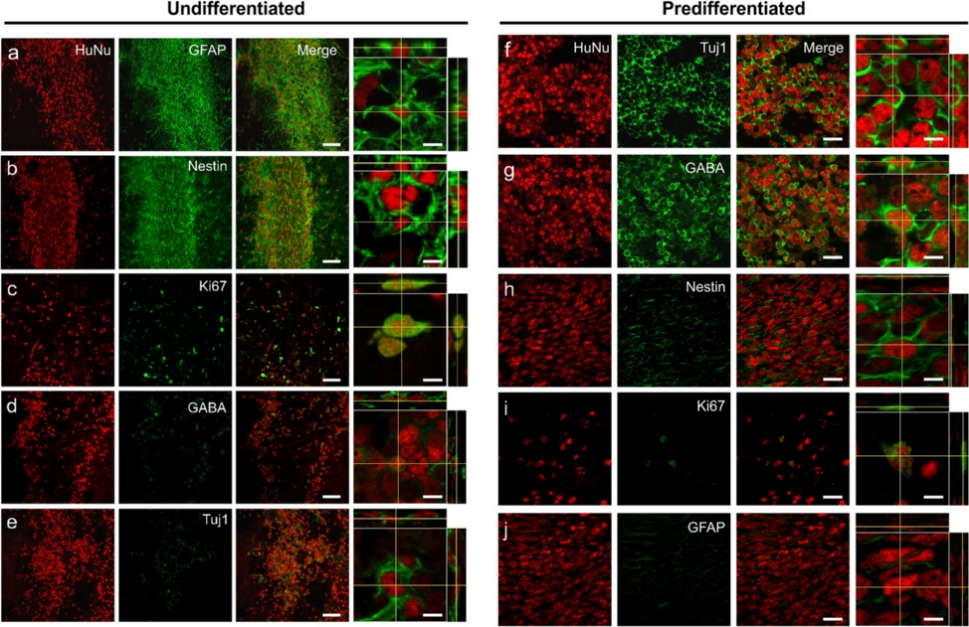
Undifferentiated hNSCs and predifferentiated cell phenotypes in vivo*.* Confocal immunofluorescent photomicrographs of undifferentiated hNSCs at 28 days post transplant within cortical border regions with orthogonal reconstructions. Undifferentiated hNSCs maintained expression of HuNu (*red*) and colabeled mainly with GFAP (**a**), Nestin (**b**), and Ki67 (**c**), with little expression of GABA (**d**) and Tuj1 (**e**) (*green*). Immunofluorescent confocal images of predifferentiated cells 28 days post transplant within the stroke-damaged cortex maintained expression of HuNu (*red*) colabeled with Tuj1 (**f**), GABA (**g**), Nestin (**h**), and Ki67 (**i**) (*green*), with lack of colocalization with GFAP (*green*) (**j**). Orthogonal reconstructions from confocal *z*-series are presented as viewed in *x*–*z* (top) and *y*–*z* (right) planes. HuNu immunoreactivity of undifferentiated hNSCs was observed completely surrounded by GFAP and Nestin, while HuNu immunoreactivity of predifferentiated cells was observed completely surrounded by Tuj1 and GABA. Scale bar: (**a**–**e**) 50 μm, orthogonal images 5 μm; (**f**–**j**) 20 μm, orthogonal images 5 μm. *GABA* gamma-aminobutyric acid, *GFAP* glial fibrillary acidic protein, *HuNu* human specific nuclear antigen, *Tuj1* β-III tubulin

Confocal image analysis 28 days post transplant revealed undifferentiated SVZ-hNSCs mainly expressed markers for GFAP and Nestin (Fig. 4a, b), with many cells also positive for Ki67 (Fig. 4c). Some undifferentiated SVZ-hNSCs were also found to express GABA and Tuj1 (Fig. 4d, e). In contrast, predifferentiated cells mainly expressed Tuj1 and GABA (Fig. 4f, g) with little colabeling with Nestin and Ki67 (Fig. 4h, i) and a distinct lack of GFAP expression (Fig. 4j).

**Fig. 5:**
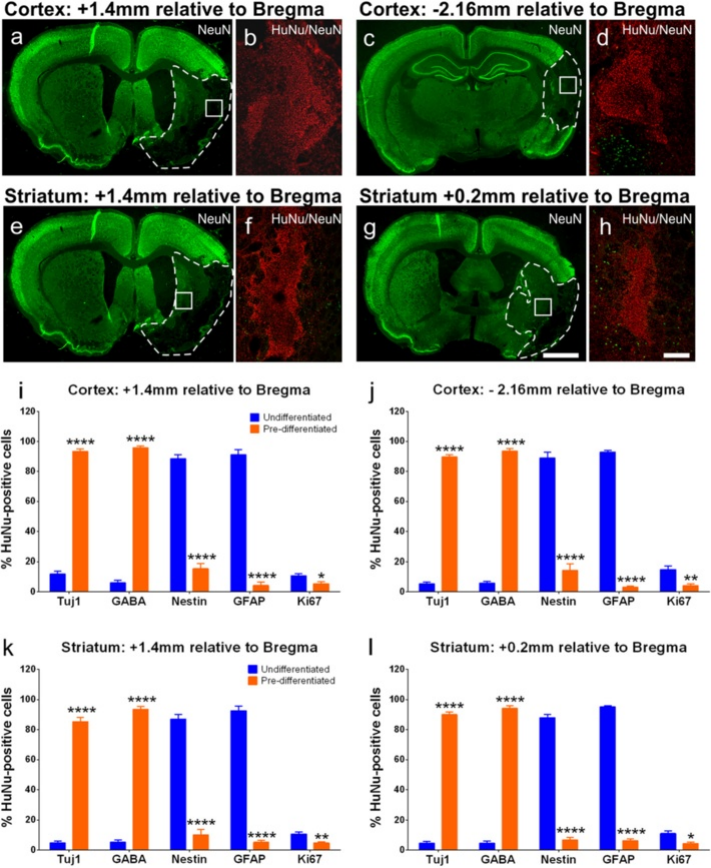
Phenotypic profile of transplanted undifferentiated hNSCs versus predifferentiated cells. Coronal sections immunostained with NeuN (*green*) highlight undamaged brain regions with lack of staining within stroke affected areas (*white dotted line*) at cortical graft sites +1.4 mm and –2.16 mm relative to the bregma (**a**, **c**) and striatal graft sites +1.4 mm and +0.2 mm relative to the bregma (**e**, **g**); *white boxes* depict graft location. Representative images of graft sites stained with HuNu (*red*) and NeuN (*green*) (**b**, **d**, **f**, **h**) from regions highlighted by *white boxes* (**a**, **c**, **e**, **g**). Numbers of HuNu-positive cells coexpressing Tuj1, GABA, Nestin, GFAP, and Ki67 from undifferentiated hNSC-treated (*n* = 7) and predifferentiated cell-treated (*n* = 7) animals from cortical graft sites; +1.4 mm (**i**) and –2.16 mm (**j**) relative to the bregma; and striatal graft sites +1.4 mm (**k**) and +0.2 mm (**l**) relative to the bregma. Numbers of cells are presented as a percentage of the total number of HuNu-positive cells counted. Data are mean ± SEM. ***P <0.001, ****P <0.0001 compared with undifferentiated hNSC counts (two-way ANOVA with Bonferroni post test). Scale bar: (**a**, **c**, **e**, **g**) 2000 μm, (**b**, **d**, **f**, **h**) 200 μm. *GABA* gamma-aminobutyric acid, *GFAP* glial fibrillary acidic protein, *HuNu* human specific nuclear antigen, *NeuN* neuron specific nuclear antigen, *Tuj1* β-III tubulin

Immunofluorescent NeuN staining was used to confirm positioning of graft sites within the infarcted region for stereological analysis across all groups receiving SVZ-hNSC transplants (Fig. 5a–h). Cell counts within each site revealed that there were significantly less cells that expressed Tuj1 (6.6 ± 1.3 %; *P* <0.0001) and GABA (5.4 ± 1.4 %; *P* <0.0001) for undifferentiated SVZ-hNSC grafts compared with predifferentiated cell grafts (Tuj1: 89.6 ± 1.9 %; GABA: 94.3 ± 1.6 %) across all transplant sites (Fig. 5i–l, ANOVA). Conversely, there was a significantly greater proportion of undifferentiated SVZ-hNSCs that expressed markers for GFAP (93.0 ± 1.9 %; *P* <0.0001), Nestin (88.1 ± 2.8 %; *P* <0.0001), and Ki67 (11.9 ± 1.6 %; *P* <0.05) compared with transplanted predifferentiated cells (GFAP: 4.7 ± 1.3 %; Nestin: 11.6 ± 3.3 %; Ki67: 4.6 ± 1.1 %) (Fig. 5i–l).

**Fig. 6:**
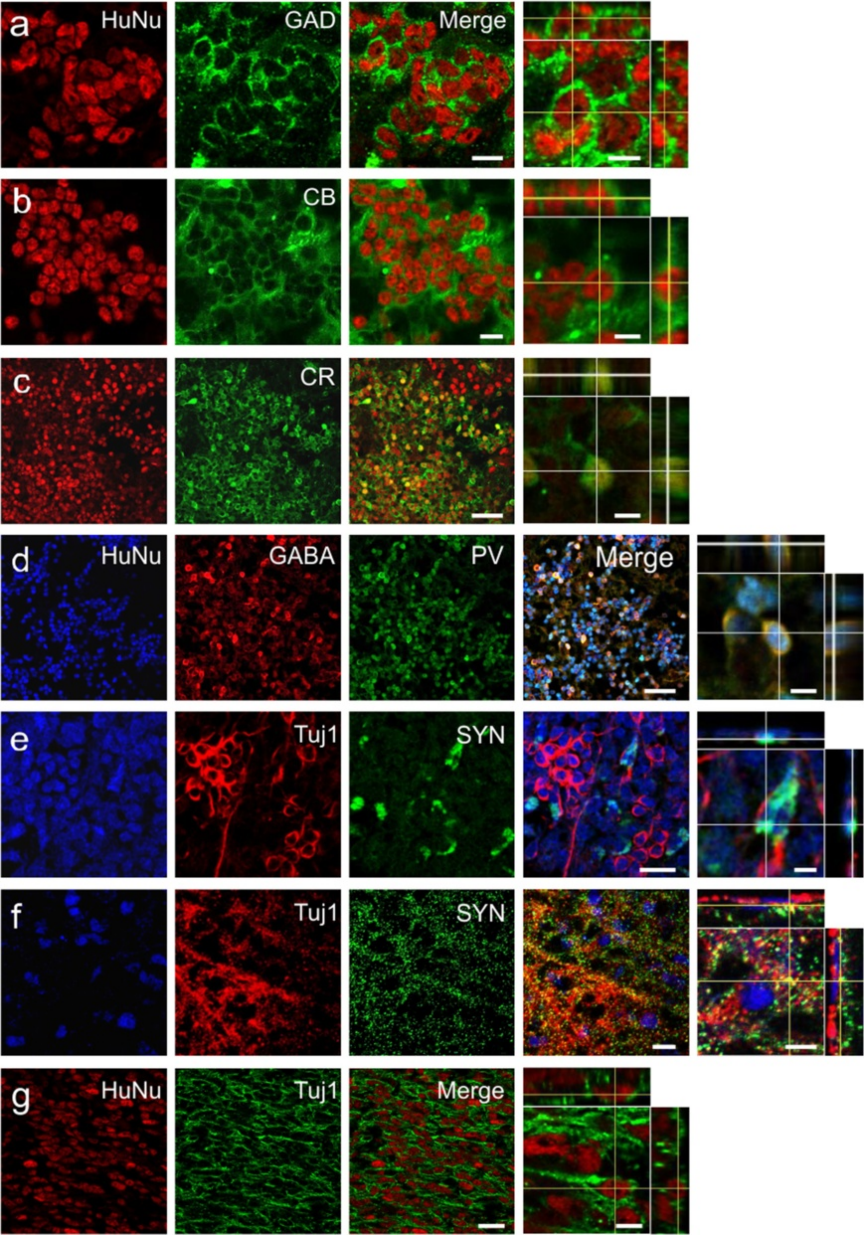
Further maturation of predifferentiated cells 28 days post transplant. Confocal photomicrographs of predifferentiated cells 28 days post transplant within the stroke-damaged brain expressed HuNu (*red*) double labeled with either GAD65/67 (*green*) (**a**), calbindin-D28k (*CB*; *green*) (**b**), or calretinin (*CR*, *green*) (**c**). Predifferentiated cells expressing HuNu (*blue*) triple-labeled with parvalbumin (*PV*, *green*) and GABA (*red*) (**d**), or triple-labeled with Tuj1 (*red*) and presynaptic vesicle protein synaptophysin (*SYN*, *green*) within the cortical core (**e**) and border (**f**) regions. Some predifferentiated cells expressing HuNu (*red*) grafted to border regions appeared to extend long neurites expressing Tuj1 (*green*) (**g**). Orthogonal reconstructions from confocal *z*-series are presented as viewed in *x*–*z* (top) and *y*–*z* (right) planes. Scale bar: (**a**, **b**) 10 μm, orthogonal image 5 μm; (**c**, **d**) 40 μm, orthogonal image 5 μm; (**e**, **f**) 10 μm, orthogonal image 5 μm; (**g**) 20 μm, orthogonal image 5 μm. *GABA* gamma-aminobutyric acid, *GAD* glutamate decarboxylase 65&67, *HuNu* human specific nuclear antigen, *Tuj1* β-III tubulin

**Fig. 7:**
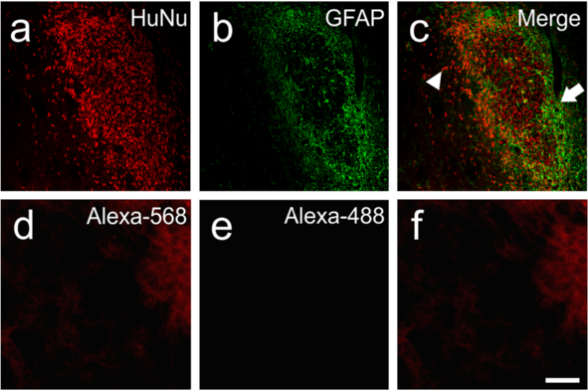
Predifferentiated cells grafted to the core infarct in vivo. Predifferentiated cell graft (HuNu, *red*) (**a**) located within the infarcted core region was surrounded by GFAP-positive astrocytes on one side (*green*) (**b**) that form the glial scar border (*arrow*) and merge (**c**), with dispersion of HuNu-positive cells observed in the core furthest away from the scar (*arrowheads*). Negative control; omission of primary antibodies (**d**–**f**). Scale bar: (**a**–**f**) 100 μm. *GFAP* glial fibrillary acidic protein, *HuNu* human specific nuclear antigen

Further confocal analysis revealed that predifferentiated SVZ-hNSC grafts expressed GAD65/67 (Fig. 6a), as well as the intracellular CBPs CB (Fig. 6b), CR (Fig. 6c), and PV (Fig. 6d). In addition, predifferentiated SVZ-hNSCs were observed to express SYN that was mostly localized to the cell body of cells transplanted into the core infarct region (Fig. 6e), but was also additionally observed along the cytoskeleton of cells that were transplanted into sites outside of the infarct within the peri-infarct territory (Fig. 6f). Predifferentiated SVZ-hNSC grafts located within the peri-infarct territory appeared to extend long neurites revealed by HuNu and Tuj1 immunostaining (Fig. 6g). Grafts located within the core infarct were surrounded by the glial scar consisting of densely packed GFAP-positive astrocytes, with limited dispersion of SVZ-hNSCs in closest proximity to the scar (Fig. 7a–c). Parallel negative control experiments omitting primary antibodies depict a low level of autofluorescence within the core infarct (Fig. d–f).

##### Effect of cell transplantation on the neurogenic niche

**Fig. 8:**
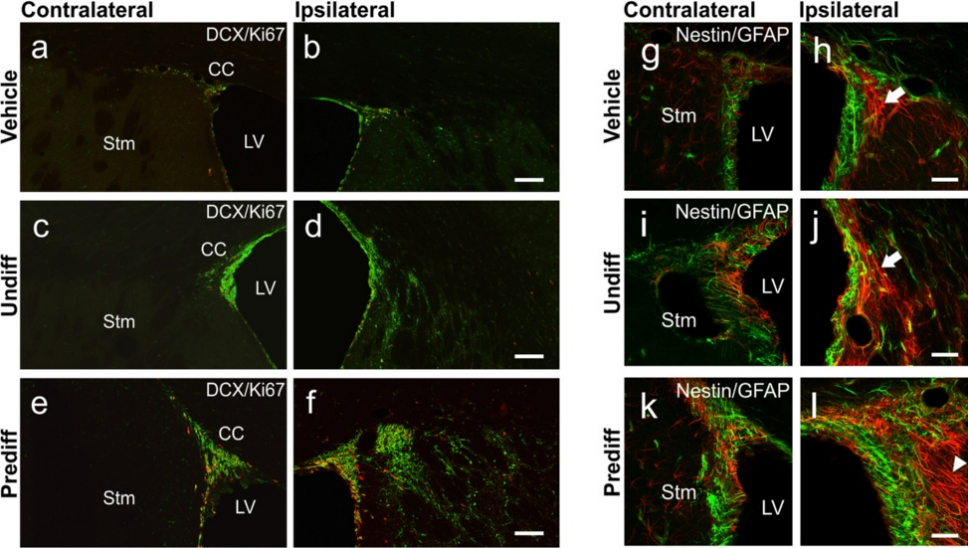
Effect of treatment on endogenous neurogenesis and radial glial populations within the SVZ. Immunofluorescent images of proliferating cells (Ki67; *red*) and migrating immature neuroblasts (DCX; *green*) within the contralateral and ipsilateral SVZ of vehicle-treated (**a**, **b**), undifferentiated hNSC-treated (**c**, **d**), and predifferentiated cell-treated (**e**, **f**) animals. Nestin (*green*; *arrowheads*) and GFAP (*red*) immunopositive cells (coexpression giving a yellow appearance; *arrows*) within the contralateral and ipsilateral SVZ of vehicle-treated (**g**, **h**), undifferentiated treated (**i**, **j**), and predifferentiated treated (**k**, **l**) animals. All images were taken at the same anatomical location from animals with similar infarct volumes. Scale bar: (**a**–**f**) 500 μm, (**g**–**l**) 50 μm. *CC* corpus callosum, *DCX* doublecortin, *GFAP* glial fibrillary acidic protein, *LV* lateral ventricle, *Prediff* predifferentiated treated, *Stm* Striatum, *Undiff* undifferentiated hNSC treated

To investigate the effects of SVZ-hNSC transplant on endogenous recovery mechanisms we examined changes within the neurogenic niche in response to stem cell transplant. Confocal analysis revealed an apparent increase in the number of Ki67-positive proliferating cells within the ipsilateral SVZ of rats that received predifferentiated SVZ-hNSCs in comparison with rats that received undifferentiated SVZ-hNSCs or vehicle controls (Fig. 8a–f). In particular, immunofluorescent labeling of cells within the SVZ revealed an apparent increase in the number of newly generated DCX-positive neurons in rats with predifferentiated SVZ-hNSC transplants in comparison with the contralateral SVZ, and rats with undifferentiated hNSC transplants or vehicle controls (Fig. 8b, d, f). Further analysis of immunolabeled cells within the SVZ revealed an apparent increase in the number of GFAP-positive radial glial cells with long processes directed towards the infarct that did not colocalize with Nestin in animals that received predifferentiated SVZ-hNSC transplants compared with the contralateral SVZ, and compared with the other treatment groups (Fig. 8g–l). Furthermore, cells identified within the neurogenic niche of the SVZ were not immunopositive for HuNu, a marker of hNSCs.

## Discussion

The use of undifferentiated stem cells for treatment of neurological diseases including stroke have undergone intense investigation in preclinical models [49–51]. Undifferentiated stem cells are thought to provide trophic factor support to injured or “depressed” neural circuitry and respond to host microenvironmental cues by migrating and differentiating into various cell types to influence functional recovery. An alternative approach to cell-based therapies, however, includes predifferentiating hNSCs prior to transplant to improve functional outcomes in animal models of Huntington’s disease, Parkinson’s disease, and neuropathic pain models [25, 26, 28].

Here we report the first evidence in a model of stroke for accelerated improvement in motor function following transplantation of predifferentiated human GABAergic hNSCs compared with undifferentiated hNSCs. We demonstrate that GABAergic neuronal transplants survive within the stroke-affected rat brain, mature, and extend processes with evidence of synaptogenesis and calcium signaling events associated with neurotransmission. Moreover, rats receiving predifferentiated cells showed evidence of increased endogenous neurogenesis, with immature neurons seen extending from the SVZ towards the damaged striatum. In contrast, undifferentiated SVZ-hNSC transplants predominantly differentiated into astrocytes and appeared to contribute to scar formation within the peri-infarct border zone. Our results suggest that differentiation of hNSCs into a neuronal phenotype prior to transplant may be a favorable strategy for in vivo cell restoration and improved motor function after stroke.

### In vitro cell phenotyping

In vitro differentiation of SVZ-hNSCs prior to transplantation at day 7 revealed a highly enriched population of GABAergic neurons that expressed Tuj1, GABA, and GAD [29]. The expression of both GAD isoforms (65 and 67) confirmed functional differentiation of these cells with an ability for neurons to produce and release GABA [52, 53] and excluded the possibility of GABA immunoreactivity resulting from in vitro uptake [28]. The decreased expression of markers Nestin, Ki67, and SOX2 with in vitro differentiation was consistent with phenotypic GABAergic conversion. Predifferentiated cell cultures comprised few Ki67-positive cells indicating the majority of cells had undergone differentiation. In contrast, examination of undifferentiated hNSCs revealed a larger population of Nestin, Ki67, and SOX2 immunopositive cells. Furthermore, previous in vitro analysis of predifferentiated hNSCs using our GABAergic predifferentiation protocol has already shown that differentiation does not result in the expression of markers for oligodendrocytes, D2 dopamine receptors, or postsynaptic glutamate receptors. It does, however, result in downregulation of precursor cell associated markers and leads to the majority of hNSCs expressing GABAergic neuronal phenotypic markers [28]. Taken together, differentiation in vitro results in downregulation of stem cell markers and an upregulation of neuronal markers where the majority of predifferentiated cells displayed a GABAergic neuronal phenotype [29].

#### Effect of cells on functional recovery

Treatment with differentiated GABAergic hNSCs accelerates functional recovery when compared with rats treated with undifferentiated hNSCs. In particular, neurological deficit scores were improved at earlier times after stroke in comparison with undifferentiated hNSC-treated rats. Recovery observed in GABAergic cell-treated rats 28 days post transplant was consistent with prestroke neurological scores. Unlike other treatments, GABAergic cell treatment resulted in statistically improved forelimb asymmetry, comparable with prestroke asymmetry. These results show that differentiation into a GABAergic cell lineage prior to transplant has beneficial effects on recovery for both the cortical and subcortical motor systems.

#### Transplant survival and migration

Only rats with the same stroke severity scores were included in these experiments, which resulted in little variation detected between infarct volumes across treatments. As such we were able to assess cell responses to the host microenvironment without having to take into account variations in brain injury between groups [32].

Despite the aforementioned findings, a comparison of cell survival 28 days post transplant between treatment groups was difficult due the apparent migration of undifferentiated cells away from the graft site. In our study, only 16 % of undifferentiated hNSCs were detected within striatal grafts and only 4 % within cortical grafts 28 days post transplant. Undifferentiated hNSCs favor less hostile environments and have previously been reported to migrate towards border regions [54, 55], which could account for the low cell numbers detected in the original grafts. For this reason, the peri-infarct territory is currently the target area for transplantation because it is thought to be less hostile and may provide a more supportive extracellular matrix for cell grafts to anchor [22, 37, 56]. In contrast, we now show for the first time that a greater percentage of cells can be retained in the target region if stem cells are predifferentiated. Approximately 30 % of GABAergic cell transplants were still detected in cortical and striatal graft sites even within the severely damaged brain 28 days post transplant. These cells were not in a state of decay since they did not stain positive for apoptotic markers Casp3 or TUNEL stain.

Previous reports suggest that between 0.5 and 15 % of exogenously grafted cells survive in the core infarct and only 20–30 % survive in locations distant to the infarct [18–22]. We now show that cell retention following transplant can be improved through predifferentiation, albeit some cell loss is probably necessary since pruning and refinement of cell numbers to facilitate development of efficient networks is an important process during development [57, 58]. Our findings may support the delivery of fewer cells while still achieving therapeutic outcomes.

The question remains: how do GABAergic cells survive within the severely damaged brain? The answer may relate to trophic factor support provided from blood vessels detected within and surrounding grafts [59–61]. Angiogenesis occurs as early as 3 days after ET-1 stroke [62] and is thought to provide a platform for brain repair. Cells grafted to sites of revascularization after stroke might be expected to do better [62–64]. Current clinical trials favor delaying cell-based treatments until behavioral deficits reach a steady plateau and the microenvironment is less aggressive [11, 65]. Unfortunately, delaying treatment also allows time for newly formed vasculature to regress [66]. Our results suggest that cell-based therapies may benefit from a developed microvascular bed for optimizing survival and influence of exogenous grafts on functional recovery. Timing cell-based therapy within weeks after stroke, when standard therapeutic procedures have been exhausted and relative stabilization of the infarct has occurred, may be a more suitable option for intervention.

#### Posttransplant histology

Immunohistochemical analysis revealed predominant differentiation of hNSCs into GFAP-positive astrocytes 28 days post transplant in all brain regions targeted. Astrocytes are becoming recognized as a restorative therapeutic target for brain injury, including stroke, because their phenotype can mediate aspects of brain integrity, neuronal cell death, and repair [67]. Astrocyte activation and reactive astrogliosis can result in both beneficial and deleterious responses, and is dependent on the intensity and hostility of the stroke-damaged environment [32, 67, 68]. In the ET-1 stroke model, reactive astrogliosis and glial scarring is established by 14 days [62] and is a major obstacle to brain repair and functional recovery [69, 70]. For this reason we chose not to transplant cells into an established glial scar, but rather targeted transplant prior to scar formation. Nonetheless, hNSCs that were not predifferentiated prior to transplant were found to be localized within the glial scar by 28 days and intensely stained with GFAP, indicating posttransplant differentiation. Undifferentiated hNSCs also displayed a significantly greater proliferative capacity as indicated by Ki67 immunoreactivity compared with predifferentiated cells, confirming their capacity for self-renewal and expansion after transplant. Interestingly, a small subpopulation of undifferentiated hNSCs spontaneously differentiated into Tuj1 and GABA-expressing neurons by 28 days, demonstrating their ability to enter a neuronal lineage despite a greater propensity towards glial cell formation [55, 56].

In contrast to the above, immunohistochemical analysis of predifferentiated GABAergic cell transplants revealed robust expression of Tuj1, GABA, and GAD within the cell bodies, confirming their GABAergic neuronal phenotype 28 days post transplant, even in the severely damaged brain. Predifferentiated cells displayed low levels of proliferative potential, without aberrant transdifferentiation. Furthermore, they did not revert back to an undifferentiated state, suggesting these cells could be a safe source for transplantation. As such, predifferentiating cells into neurons prior to transplant may provide a better treatment option by reducing the risk of contributing to glial scar and tumor formation [71]. Future studies investigating the effects of undifferentiated hNSC or predifferentiated cell transplants on non-neuronal host cells would be of great interest to determine the extent of their influence and thus provide a well-rounded perspective of their potential.

#### Neuronal repopulation and trophic support for functional recovery

How GABAergic transplants improve the recovering brain is yet to be fully determined. GABAergic neurons are the principal inhibitory cells of the mammalian central nervous system and occur throughout the brain, and GABA regulates the proliferation of neural progenitor cells [72, 73], migration [74], and other aspects of neurogenesis including differentiation [75, 76], neurite formation [77], and synaptogenesis [78]. Possible mechanisms of recovery following stroke injury therefore include neuronal repopulation to bridge the gap between damaged circuits, rescue or reactivation of dormant but surviving circuitry, and trophic influences on endogenous neurogenesis and neuronal reconstruction.

In our study, GABAergic cells transplanted into the striatum resulted in >30 % new neurons expressing immunohistochemical markers that are typically expressed by striatal GABAergic medium-sized spiny projection neurons and aspiny interneurons, being positive for GABA, GAD, Tuj1, and CBPs that are essential components for restoring neurotransmission [79–83]. Whilst grafts to the core infarct were clearly isolated by the glial scar barrier by 28 days, grafts within the border zone appeared to be better positioned to influence host circuitry and promote recovery. Future studies using anterograde and retrograde labeling would be required to substantiate connectivity of graft cells to host neural systems [84].

GABAergic cell transplants also upregulated markers that were absent at the time of transplant, indicating further maturation in vivo to potentially aid recovery. In our study, only predifferentiated cells expressed SYN, a presynaptic vesicle protein, suggesting increased synaptic vesicle formation with potential roles in neurotransmission and plasticity [85–87]. The localization of SYN to the cellular cytoskeleton of grafts within the peri-infarct territory could indicate a more advanced state of maturation compared with grafts within the core infarct. Furthermore, only predifferentiated cells were observed to express the intracellular CBPs CB, CR, and PV, potentially indicating calcium signaling events associated with neurotransmission, transmitter release, and plasticity [88, 89]. Overall, paracrine delivery of GABA, upregulated SYN, and CBP expression may restore neurotransmission and promote plasticity to improve behavioral outcomes.

Another possible outcome of GABAergic transplant is the awakening of depressed pathways known to lie dormant following stroke [90–92], either through restoration of neurotransmission or graft-induced enhancement of neurogenesis to stimulate pathways and influence repair. Trophic and chemoattractive functions exerted by interneurons during development through the release of depolarizing GABA may be recapitulated by GABAergic transplants to promote proliferation/migration of endogenous progenitor cells, provide positional cues to migrating cells including immature neurons, and influence synapse maturation [52, 93, 94]. This is concordant with the human GABAergic transplants in our study potentially being immature and therefore excitatory through release of depolarizing GABA as the switch to an inhibitory phenotype that only occurs late in the first postnatal year in humans [95]. Furthermore, within the ipsilateral SVZ, only predifferentiated cell-treated animals displayed GFAP-positive radial glial cells with long processes known to function in guiding immature neurons during neurogenesis [96]. Although the endogenous neurogenic response purportedly increases 7–14 days post stroke and returns to normal beyond this time [97], the apparent increase in activity within the neurogenic niche in our study 35 days post stroke suggests a trophic influence from transplanted GABAergic cells. In addition, the low neurogenic response observed in vehicle-treated animals 35 days post stroke supports previous reports demonstrating basal neurogenesis levels beyond 2 weeks post stroke in rats [97]. Trophic factors secreted from migrating endogenous progenitor cells and immature neurons, or GABAergic transplants themselves, could therefore act to stimulate rewiring or awakening of host circuits, enabling remaining healthy tissue to restore function of lost connections [92, 98, 99].

## Conclusion

Findings from this study demonstrate predifferentiating SVZ-hNSCs prior to transplantation as a novel strategy to accelerate functional recovery after cerebral ischemia. In contrast, undifferentiated hNSC transplants have the potential to delay or hinder functional improvements through contributions to further scar formation. Neuronal repopulation through GABAergic neuronal transplants may achieve greater viability in a clinical setting by targeting a smaller ischemic region, as seen in lacunar strokes [100, 101]. However, GABAergic transplants also show promise for targeting severe stroke through production of GABA, synaptogenesis, and calcium signaling events associated with neurotransmission, to bridge the gap between damaged pathways, enhance plasticity, or rescue depressed circuitry to re-establish function. Alternatively, known trophic effects of depolarizing GABA during development may be recapitulated in our study to enhance endogenous neurogenesis, reactivate circuitry, and promote plasticity for functional recovery in severe strokes with widespread damage. The principles behind differentiation of hNSCs into a desired neuronal cell type may be a favorable alternative for treating stroke.

## Abbreviations

ANOVA: Analysis of variance
BDNF: Brain-derived neurotrophic factor
bFGF: Basic fibroblast growth factor
BSA: Bovine serum albumin
Casp3: Cleaved caspase-3
CB: Calbindin-D28k
CBP: Calcium binding protein
CR: Calretinin
DAPI: 4′,6-Diamidino-2-phenylindole
DCX: Doublecortin
DMEM: Dulbecco’s modified Eagle’s medium
EGF: Epidermal growth factor
ET-1: Endothelin-1
GABA: Gamma-aminobutyric acid
GAD: Glutamate decarboxylase 65&67
GFAP: Glial fibrillary acidic protein
hNSC: Human neural stem cell
HuNu: Human specific nuclear antigen
MCA: Middle cerebral artery
NeuN: Neuron specific nuclear antigen
NGS: Normal goat serum
NSC: Neural stem cell
PBS: Phosphate-buffered saline
PFA: Paraformaldehyde
PV: Parvalbumin
SEM: Standard error of the mean
SVZ: Subventricular zone
SYN: Synaptophysin
Tuj1: β-III Tubulin
TUNEL: Terminal transferase-mediated dUTP nick end-labeling
vWF: Von Willebrand factor
